# Use of Direct Hemoperfusion with Polymyxin B-Immobilized Fiber for the Treatment of Septic Shock Complicated with Lemierre Syndrome Caused by *Fusobacterium necrophorum*

**DOI:** 10.1155/2019/5740503

**Published:** 2019-07-30

**Authors:** Yoshiyuki Kodama, Gaku Takahashi, Shigenori Kan, Takayuki Masuda, Yoriko Ishibe, Rise Akimaru, Maria Nonoguchi, Yoshihiro Inoue

**Affiliations:** Department of Critical Care, Disaster and General Medicine, School of Medicine, Iwate Medical University, 19-1 Utimaru, Morioka City, Iwate 020-8505, Japan

## Abstract

We report a case of septic shock treated with PMX-DHP that was complicated with Lemierre syndrome caused by *Fusobacterium necrophorum*. The patient was a 31-year-old man who was initially diagnosed with influenza. He received treatment; however, because his symptoms gradually worsened, he was transported to our hospital 10 days following his diagnosis. His initial examination revealed symptoms of respiratory distress and an altered level of consciousness. Based on laboratory and imaging results, it became clear that he suffered from septic shock of unknown etiology, disseminated intravascular coagulation, and acute renal and respiratory failure. We initiated treatment with vasopressors, an antibacterial agent, immunoglobulins as well as an appropriate ventilator management; however, his unstable circulatory condition continued. As soon as PMX-DHP was initiated, 2 days following admission to the ICU, his circulatory instability normalized. *F. necrophorum* was ultimately detected after a culture examination, and contrast-enhanced computed tomography revealed a jugular vein thrombus, which led to the diagnosis of Lemierre syndrome. The patient's condition gradually improved, and he was discharged from the ICU after 19 days.

## 1. Introduction

*Fusobacterium* spp. is an anaerobic gram-negative bacillus present in the oral cavity and gastrointestinal tract [1, 2]. However, this organism rarely causes Lemierre syndrome with thrombophlebitis of the internal jugular vein in healthy young individuals [3]. Although the incidence rate has recently been reported to be 0.6–2.3 cases per million [4], the mortality rate is as high as 4–22% [5]. In this study, we report a case of polymyxin B-immobilized fiber column direct hemoperfusion for the treatment of septic shock complicated with Lemierre syndrome caused by *F. necrophorum*.

## 2. Case Presentation

A 31-year-old man, with no significant medical history, presented with a chief complaint of fever and altered level of consciousness. On April XX, 2018, cold symptoms first appeared. He was diagnosed with influenza by the family physician, and was treated with oseltamivir. However, the symptoms progressed, and it became difficult for him to move after 10 days; therefore, he was admitted to our hospital. His initial examination revealed symptoms of respiratory distress and altered consciousness. Based on blood tests and imaging, he was diagnosed with septic shock of unknown origin. Furthermore, disseminated intravascular coagulation and acute renal and respiratory failure complicated his course, and he was admitted to the ICU.

Upon admission to the ICU, the patient was 183 cm tall and weighed 65.1 kg. He had a Glasgow Coma Scale score of E4V4M6, pulse rate of 130 bpm, blood pressure of 60/45 mmHg, respiratory rate of 30/min, SpO_2_ of 93% on 10 L O_2_, and fever of 39.6°C. There was no apparent swelling or redness in the neck. Physical examination revealed that heart sounds were normal and moist rales were detected in the lungs, but there were no anomalies in the abdomen, limbs, or skin.

**Figure 1:**
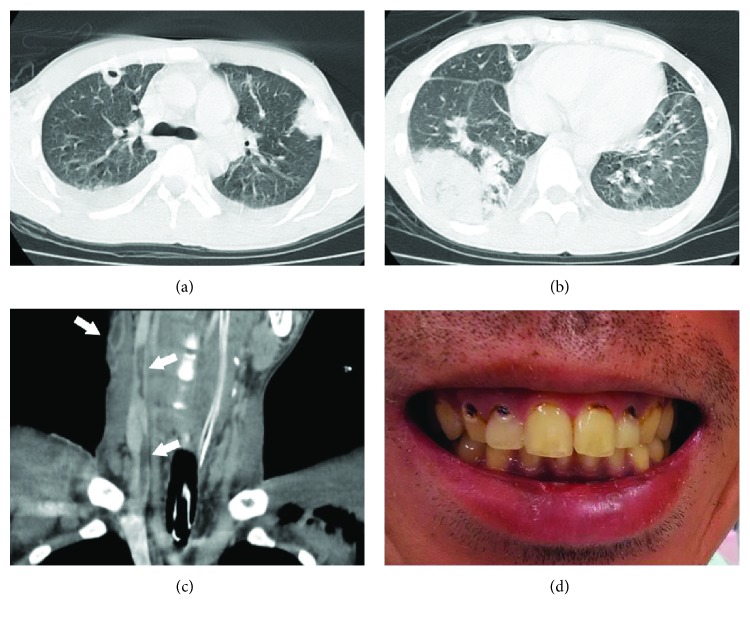
(a, b) Computed tomography undertaken upon hospital admission (pulmonary window setting). Multiple bilateral peripheral pulmonary masses with cavitation and pleural effusion can be observed. (c) Contrast-enhanced computed tomography. Thrombosis of the right internal and external jugular vein can be observed (arrows). (d) Intraoral examination. Periodontal disease and multiple caries are revealed in the oral cavity.

Multiple nodular shadows and infiltrates were observed in both lung fields on chest X-ray. Chest computed tomography (CT) revealed cavitary lesions in both lung fields, and the nodules primarily occurred under the pleura (Figure 1(a) and 1(b)). A bilateral pleural effusion was noted. The ejection fraction was 63% on transthoracic cardiac ultrasound examination. Wall motion was normal and no obvious vegetations were observed. There were no irregular findings on head and abdominal CT.

Laboratory findings upon admission are given in Table 1 and include the following: WBC: 17880/*µ*L; BUN: 85.1 mg/dL; Cre: 2.76 mg/dL; Platelet count: 24000/*µ*L; CRP: 25.75 mg/dL; P-SEP: 3551.0 pg/mL; PCT: 77.21 ng/mL; Endotoxin: 29.1 pg/mL; PT-INR: 1.52; DD: 1.9 *µ*g/mL, AT3: 58%; HbA1C: 5.8%; Glu: 121 mg/dL; arterial blood gas (10 L oxygenated reservoir) pH: 7.509; pO_2_: 73.9 mmHg; pCO_2_: 26.9 mmHg; HCO_3_^−^: 20.5 mol/L; BE: −0.2 mmol/L; Lactate: 3.4 mmol/L; Influenza test: A(−), B(−), and HIV: (−); Sequential organ failure assessment score: 15; Acute physiology and chronic health evaluation II (APACHE II) score: 32; and Acute DIC score: 5.

**Figure 2:**
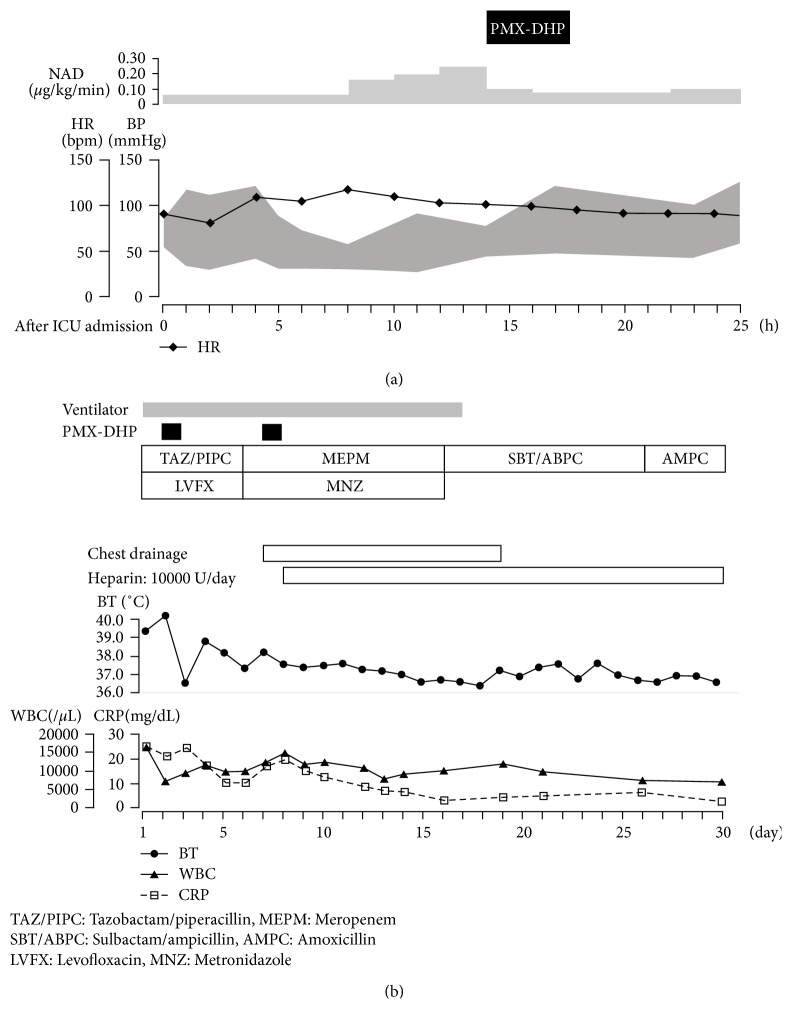
Clinical course. (a) Initial 24 h in the ICU. (b) After 30 days of hospitalization.

The patient's post-hospitalization course is given in Figure 2. Upon admission to the ICU, he was intubated and placed on a ventilator. Based on Sepsis-3 [6], he was diagnosed with septic shock, and we administered a continuous infusion of noradrenaline (NAD). Although the infection source and causative bacterial species were unknown, the likelihood of the presence of an endotoxin was high; therefore, tazobactam/piperacillin and levofloxacin were administered. Antithrombin and gabexate mesylate were administered for septic DIC. Despite a high dose of NAD, circulatory insufficiency remained significant; 2 days after admission to the ICU, PMX-DHP (PMX-20R) was administered for about 3 h. His blood pressure began to immediately rise; once his blood pressure was stable, about 3 h after initiation of the infusion, the dosage of NAD was gradually decreased.

*F. necrophorum* (Table 1) was detected in the blood culture on the 6th day after admission to the ICU. On the same day, cervical contrast-enhanced CT, neck echocardiography, and intraoral examination were performed. Cervical contrast-enhanced CT and neck echocardiography revealed a thrombus in the right internal and external jugular veins, and emboli were present in both lung fields on chest CT (Figure 1(c)). Intraoral examination revealed periodontal disease and multiple caries in the oral cavity (Figure 1(d)). These findings led to the diagnosis of Lemierre syndrome caused by *F. necrophorum*. With continued oral care and antimicrobial treatment, his respiratory condition gradually improved, and he was removed from the ventilator on the 16th day after admission to the ICU, and moved to the general ward on the 20th day.

## 3. Discussion

In Lemierre syndrome, thrombophlebitis of the internal jugular vein is caused by acute pharyngitis and abscess in the amygdala. In severe cases, bacterial emboli from the internal jugular vein spread to the lung, liver, kidney, bone, and joints. Lemierre syndrome is more common in healthy young individuals, and the male- to-female ratio was 1 : 2 [7]. In addition, among the 114 cases of Lemierre syndrome, it has been reported that the proportion of the 1st decade to 3rd decade was 79% [7]. Even in this case, it was an inherently healthy young person.

Notably, *F. necrophorum* has been identified as the causative species in at least 80% of patients with Lemierre syndrome [8]. In our case, in addition to periodontitis and numerous dental caries, pharyngitis caused by the influenza virus may have triggered the onset of the syndrome. Furthermore, the infection route is consistent with that reported previously [9, 10].

Lemierre syndrome is characterized by (1) a history of recent oropharyngeal infection, (2) clinical or radiological evidence of internal jugular vein thrombosis, and (3) isolation of anaerobic pathogens, mainly *Fusobacterium necrophorum* [11]. The patient did fulfill these characteristics.

For several days after admission to the ICU, although the infection source and causative bacterial species were unknown, the endotoxin levels were high, and intensive care was provided based on the presumed diagnosis of septic shock from gram-negative bacteria. We believe that the initial treatment was appropriate because it took 6 days before the culture was positive for *F. necrophorum*; this suggests the diagnosis of Lemierre syndrome. To the best of our knowledge, this is the first report of PMX-DHP being used for the successful treatment of septic shock with Lemierre syndrome caused by *F. necrophorum*. In our case, the endotoxin level decreased from 6.8 pg/mL before PMX-DHP infusion to 2.6 pg/mL after infusion. In addition, his blood pressure was quite unstable prior to PMX-DHP infusion, with 74/44 mmHg before infusion to 113/47 mmHg after infusion, after which it stabilized. As a result, the infusion of NAD could be gradually decreased from 0.23 *µ*g/kg/min to 0.08 *µ*g/kg/min. *F. necrophorum* tends to form a septic thrombus by destroying red blood cells and aggregating platelets and bacteria by producing endotoxins [12]. Therefore, PMX-DHP was considered to be effective based on this mode of action.

We describe a report in which PMX-DHP was used for the treatment of septic shock complicated with Lemierre syndrome caused by *F. necrophorum*. In addition to the early use of appropriate antimicrobial therapy, PMX-DHP should be considered a supportive therapy in similar situations.

## Figures and Tables

**Table 1:** Laboratory data upon hospital admission.

*Hematology*	
WBC	17880/*μ*L
RBC	3.54 million/*μ*L
Hb	11.5 g/dL
Hct	32.9%
Plt	24000/*μ*L

*Coagulation tests*	
PT	19%
PT-INR	1.52
APTT	43.9 s
D-dimer	1.9 *μ*g/mL
AT3	58%

*Biochemistry*	
AST	66 U/L
ALT	46 U/L
LDH	472 U/L
*γ-*GTP	36 U/L
T-BIL	4 mg/dL
D-BIL	3.1 mg/dL
CK	107 U/L
TP	5.1 g/dL
Alb	2 g/dL
BUN	85.1 mg/dL
Cre	2.76 mg/dL
Na	128 mEq/L
K	3.7 mEq/L
Cl	89 mEq/L

*Serology*	
CRP	25.75 mg/dL
Presepsin	3551 pg/mL
Procalcitonin	77.21 ng/mL
Endotoxin	29.1 pg/mL
*β-*D glucan	≦5.0 pg/mL
HbA1C	5.8%
Glu	121 mg/dL
HBs-Ag	(−)
HCV-Ab	(−)
HIV	(−)

*Arterial blood gases*	
(10 L reservoir oxygen)	
pH	7.509
pCO_2_	26.9 mmHg
pO_2_	73.9 mmHg
HCO_3_^−^	20.5 mEq/L
BE	−0.2 mEq/L
Lac	3.4 mmol/L

*Urinalysis*	
Pneumococcal-Ag	(−)
Legionella-Ag	(−)

*Influenza*	
Type A	(−)
Type B	(−)

*Acid-fast*	
Smear	(−)
Culture	(−)

*Culture*	
Sputum and blood	
*Fusobacterium necrophorum*	

*MIC of F. necrophorum*	

Antibiotics	MIC

Ampicillin	0.12S
Penicillin G	0.06S
Sulbactam/ampicillin	≦0.06S
Cefmetazole	≦1S
Meropenem	≦0.25S
Levofloxacin	≦2S
Clindamycin	≦0.12S
Tazobactam/piperacillin	≦16S
